# Mass spectrometric investigations of the action of hypochlorous acid on monomeric and oligomeric components of glycosaminoglycans

**DOI:** 10.1016/j.bbrep.2023.101448

**Published:** 2023-03-02

**Authors:** Jenny Leopold, Patricia Prabutzki, Ariane Nimptsch, Jürgen Schiller

**Affiliations:** Institute for Medical Physics and Biophysics, Faculty of Medicine, University of Leipzig, Härtelstrasse 16-18, 04107, Leipzig, Germany

**Keywords:** Hypochlorous acid, Hyaluronan, Glycosaminoglycans, Monosaccharides, ESI MS, NMR spectroscopy, UV spectroscopy

## Abstract

Hypochlorous acid (HOCl) is a strong non-radical oxidant, which is generated during inflammatory processes under the catalysis of the enzyme myeloperoxidase (MPO). HOCl reacts particularly with sulfhydryl and amino acid residues but affects also many other biomolecules. For instance, the glycosaminoglycans of articular cartilage and synovial fluids (such as hyaluronan) undergo degradation in the presence of HOCl at which the native polysaccharide is fragmented into oligosaccharides in a complex reaction.

This is an initial mass spectrometry (MS)-based investigation dealing with the HOCl-induced degradation of glycosaminoglycans and the conversion of the related monosaccharides into chlorinated products. In particular, it will be shown that the reaction between HOCl and hyaluronan is slower than originally assumed and results in the generation of different products (particularly the hyaluronan monosaccharides) by the cleavage of the β-1,3/1,4-glycosidic linkages. The MS detection of chlorinated products is, however, only possible in the case of the monosaccharides. Potential reasons will be discussed.

## Highlights

• HOCl is a powerful oxidant and relevant in many inflammatory diseases.
• Hyaluronan (HA) is an important constituent of cartilage and synovial fluids.
• It is known that the molecular weight of HA decreases during inflammation.
• Mass spectrometry helps to study the pathways of glycosaminoglycan degradation.
• The glycosidic linkages are particularly fragmented by HOCl.

## Graphical abstract

**Figure:**
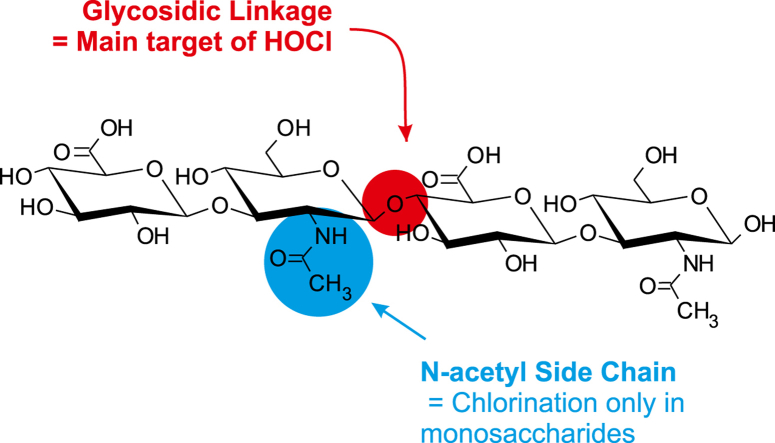

## 1 Introduction

Large numbers of polymorphonuclear leukocytes (neutrophils) occur in the synovial fluid of patients suffering from rheumatoid arthritis, which is known to be the most common inflammatory disease of joints [1,2]. It is commonly accepted that these polymorphnuclear neurophils (PMN) - or products derived thereof - contribute to the damage of articular cartilage in chronic inflammatory diseases like rheumatoid arthritis [3] and decrease the viscosity of synovial fluid - in particular by reducing the molecular weight of hyaluronic acid (HA), a major constituent (about 2–3 mg/ml) of synovial fluid [4].

Neutrophils generate potent reactive oxygen species (ROS) including superoxide anion radicals (O_2_^•-^), hydrogen peroxide (H_2_O_2_), hydroxyl radicals (HO^•^), hypochlorous acid (HOCl) and singlet oxygen (^1^O_2_) as well as different proteolytic enzymes (matrix metalloproteinases, myeloperoxidase (MPO) and elastase) and glucuronidases upon stimulation [5,6]. Although the contribution of neutrophils to rheumatic diseases is well established, detailed mechanisms of tissue damage, regulations and defense reactions remain widely unknown [7].

We have focused our main interest on neutrophil-derived HOCl due to its strongly oxidizing properties and the singularity of its generation. HOCl is generated in a MPO catalyzed reaction between H_2_O_2_ and chloride ions (Cl^−^) [8]:

H_2_O_2_ + Cl^−^ → HOCl + HO^−^

MPO is a highly abundant protein of the azurophilic granules of neutrophils [9] and constitutes about 5% of the total protein content in these cells [10]. Thus, significant amounts of HOCl can be generated under *in vivo* conditions and concentrations up to 340 μM are discussed to be of physiological relevance [11].

Detailed studies on both, the reaction of cartilage and synovial fluid polysaccharides (such as HA) with HOCl, as well as the detailed chemical mechanisms behind these reactions were barely performed. For example, it has been shown by viscosimetry and high-performance liquid chromatography (HPLC) [12] that a viscosity loss of HA solutions is induced at relatively small HOCl concentrations, while much higher concentrations of HOCl are required to cleave the glycosidic linkages within the polysaccharide under the formation of low molecular weight breakdown products. Both effects indicate that complex reaction patterns are involved in these oxidation processes but HOCl-induced effects are surely involved [13].

Additionally, the putative mechanism of the involved reactions was studied using ^1^H NMR spectroscopy [14]. HOCl reacts in the first step with the *N*-acetylglucosamine (GlcNAc) unit of HA. Two different effects could be observed: the depolymerization of HA and the cleavage of the *N*-acetyl groups to yield acetate as final product via a transient chlorinated product. This product and the derived *N-*centered free radicals were afterwards also confirmed by electron spin resonance and other methods [15]. Further oxidation products such as formate are only detectable in the presence of a large excess of HOCl. Since formate is a well-known product of HO^•^-induced carbohydrate degradation [16] this implies the conversion of HOCl into other ROS before the reaction with the carbohydrate takes place. Akeel et al. [17] studied both the kinetics and the reaction profile of the *in vitro* reaction of HOCl with either HA or heparin using spectrophotometrically- or enzymatically-based methods and found differences in dependence on the sulfate content [17].

Mass spectrometry (MS) would represent another very powerful technique to study HOCl-induced oxidation processes of oligosaccharides in more detail because it enables the monitoring of characteristic changes of the molecular weight, which occur, for instance, upon cleavage of the glycosidic linkages. Therefore, it is very surprising that there are so far (to the best of our knowledge) nearly no investigations in this field available. The only published approach [18], where electrospray ionization (ESI) MS was used to investigate the degradation of HA, was not very successful - not to mention that peroxynitrite was used as the oxidizing agent instead of HOCl. In a recent study, native, high molecular weight hyaluronan was converted into the corresponding chloramide but the focus was here on chlorinated isocyanuric acid as the chlorinating agent [19] and to a lesser extent HOCl (which was indicated as a less efficient chlorinating agent). Other groups used gas chromatography (GC)-MS which is only capable of detecting small carbohydrates subsequent to derivatization in order to enhance the volatility [20]. Therefore, the present study can be regarded as a first attempt to investigate the reactions between HOCl and selected components of HA by ESI MS. It will be demonstrated that the detection of N-chlorinated amides is difficult while cleavages of the glycosidic linkages in oligosaccharides and the generation of chloramines can be easily detected. It is important to note that HOCl is (in water) in equilibrium with free chlorine - particularly at acidic conditions [21]:

HOCl + HCl ⇆ H_2_O + Cl_2_

Therefore, there are increasing indications that Cl_2_ is involved in the HOCl-induced reactions of biomolecules as well as drugs in aqueous solution [22,23]. Since our aim is the detection of chlorinated GAG-derived mono- and oligosaccharides we will not pay major attention to these aspects and discuss the products exclusively as HOCl-induced effects.

## 2 Material and methods

### 2.1 Chemicals

**Fig. 1:**
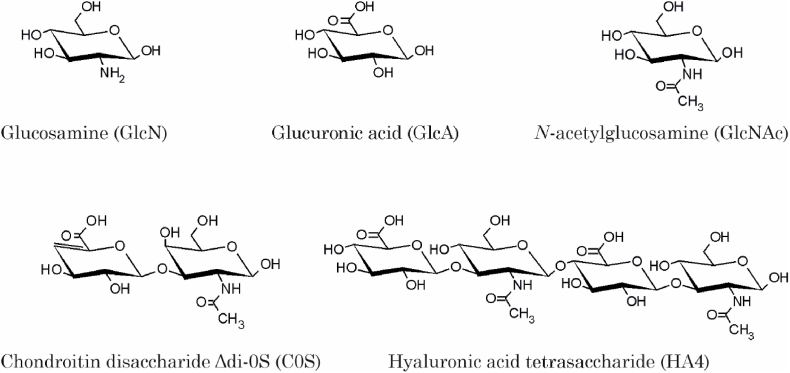
Survey of the different mono- and oligosaccharides, which were investigated in this study.

The investigated saccharides (shown in Fig. 1), namely glucosamine hydrochloride (GlcN), glucuronic acid (GlcA), GlcNAc and unsaturated chondroitin disaccharide Δdi-0S sodium salt (C0S) were purchased from Sigma-Aldrich (Taufkirchen, Germany) in the highest available purity and used without any further purification. The tetrasaccharide of hyaluronic acid (HA4) was a generous gift by Jörg Rademann (FU Berlin) and prepared as recently described [24].

Chemicals for buffer preparation (sodium hydroxide, sodium dihydrogen phosphate monohydrate, sodium acetate), solvents (acetonitrile, methanol, water), sodium hypochlorite (NaOCl; as source for HOCl) as well as the NMR standard (3-(trimethylsilyl)-1-propionic acid-d_4_) were obtained from Sigma-Aldrich.

A stock solution of NaOCl was prepared in water and its concentration spectrophotometrically determined at pH 12 (*ε*_290_ = 350 L mol^−1^ cm^−1^) each day prior to use [25]. The NaOCl stock solution was diluted to the desired working concentration with either phosphate buffer or sodium acetate buffer.

### 2.2 *In vitro* oxidation of saccharides

5 mg/mL stock solutions of carbohydrates were prepared in water and stored at −20 °C until oxidation. NaOCl solution was added to freshly prepared phosphate buffer (20 mmol/L) or sodium acetate buffer (NaOAc, 50 mmol/L) and brought to a final concentration of 20 mmol/L and pH 6. These slightly acidic pH conditions are still physiologically relevant because the pH decreases under inflammatory conditions [26]. Additionally, there is an excess of HOCl (pK = 7.53) compared to ClO^−^ at slightly acidic conditions which is important since HOCl is the more reactive species [3].

For the *in vitro* oxidation, different molar excesses of HOCl (see the Results section) were added to 30 μL of the corresponding carbohydrate solution and buffer was added to give a final volume of 600 μL. All carbohydrates were separately incubated for different time points (450 rpm, 37 °C). For the analysis by mass spectrometry, 200 μL acetonitrile was added to obtain a final carbohydrate concentration of 187.5 μg/mL. The dilution was also accompanied by a sufficient reduction of the salt content, which enabled subsequent ESI MS measurements without the need to desalt the samples.

### 2.3 Electrospray ionization mass spectrometry

ESI MS was performed on an amaZon SL ion trap (IT) mass spectrometer (Bruker Daltonics, Bremen, Germany) using nitrogen as nebulizer gas. Ion optic parameters were optimized for each natural carbohydrate prior to the measurements. The oxidized carbohydrate samples were directly introduced into the ESI source via a syringe pump at a flow rate of 2 μL/min using the following source conditions: mass range mode, enhanced resolution; dry temperature, 200 °C; nebulizer, 15.00 psi; dry gas, 10.00 L/min; HV capillary, 4.5 kV and end plate offset, 0.5 kV. These parameters enabled the unequivocal identification of the parent ion but provided reasonable intensities of fragment ions as well. Mass spectra were acquired in enhanced resolution mode (8.100 u/sec) and the ion charge control (ICC) target was set to 100.000 with a maximum acquisition time of 50 ms.

Data acquisitions and analyses were carried out by using Trap Control and Data Analysis software, respectively (Bruker Daltonics, Bremen, Germany).

### 2.4 Thin-layer chromatography (TLC)

TLC-separation was performed as previously described [27] on commercially available silica gel 60 plates (MERCK, Darmstadt, Germany). The used solvent system was n-butanol/formic acid/water (3:4:1, v/v/v) and samples (1–2 μl) were applied by using a Linomat 5 device (CAMAG, Mutenz, Switzerland). Samples were either stained with orcinol/H_2_SO_4_ or re-eluted from the TLC plate for subsequent ESI-IT MS analysis.

We have also attempted to use LC/MS in order to clarify the chemical structures of the obtained reaction products and to get rid of buffer salts and other contaminants. However, these attempts did not really result in pronounced improvements. Therefore, no major attention was paid to LC/MS. Nevertheless, all the related data are available as supplementary material.

### 2.5 UV measurements

UV measurements were performed on a Hitachi U-2000 spectrophotometer, using quartz SUPRASIL® precision cell cuvettes (light path 10 mm). The obtained data were used to compare the relative reactiveness of HOCl with the individual carbohydrates. The decrease of the HOCl/ClO^−^ concentration was determined during 3 h at λ = 290 nm in the presence and the absence of the different carbohydrates. This approach is suitable because the generated chloramines absorb at a different wavelength (λ ≈ 250–255 nm) [28] and do not lead to interferences with the reagent HOCl.

For the UV experiments, the concentration (20 mM) and the final volume (150 μL) of HOCl were kept constant. The volume of each carbohydrate solution (stock solution 1 mg/mL each) was chosen to obtain a molar ratio of 1:10 (carbohydrate:HOCl). To obtain a total reaction volume of 500 μL, which was necessary for the used cuvettes, the missing volume was made up with 20 mmol/L phosphate buffer.

### 2.6 NMR-measurements of oxidized N-acetylglucosamine

All ^1^H NMR spectra were acquired on a Bruker DRX 700 spectrometer using a 5 mm TXI probe optimized for ^1^H. All spectra were recorded at 310 K. Typically 0.40 mL carbohydrate solution was placed in a 5 mm diameter NMR tube and 50 μL of D_2_O was added to provide a field-frequency lock. The intense water signal was suppressed by the Watergate pulse sequence [29]. 32 free induction decays were accumulated with a pulse delay of 5 s between two pulses to allow complete T_1_ relaxation. Spectra were processed with a Gauss-broadening of 0.2 Hz. Chemical shifts were referenced to external sodium 3-(trimethylsilyl)-1-propionic acid (TSP) at 0.00 ppm.

The oxidation procedure of GlcNAc by HOCl for NMR measurement was as follows: a 5 M excess of HOCl (50 mmol/L) was added to 100 μL GlcNAc (10 mg/mL) and made up with phosphate buffer (50 mmol/L) to obtain a total volume of 1 mL. The incubation time was 4 h (450 rpm, 37 °C) and the reaction was stopped by adding 1 mL acetonitrile; subsequently the mixture was evaporated to dryness. Prior to the NMR experiments, phosphate buffer/D_2_O (1/1, v/v) was added to give a final concentration of 200 μg/mL of GlcNAc.

## 3 Results and discussion

Although some work on the degradation of glycosaminoglycans (GAG), such as HA, by HOCl has already been performed, there are so far nearly no investigations [19] dealing with the mass spectrometric characterization of the degradation products. In particular, this applies for the transient products such as the chloramide, which is putatively generated [14,15] in the first step of the oxidation of the GlcNAc moiety of HA. There are some major problems in the investigation of GAG oxidation by HOCl and the monitoring of these reactions by MS, which is probably the reason why such studies were only marginally performed. To ensure a reaction between the carbohydrate of interest and HOCl, the pH should be slightly acidic because HOCl (as the reactive agent) must be in excess over NaOCl. Since many common organic buffers (such as 4-(2-hydroxyethyl)-1-piperazineethanesulfonic acid, HEPES) also react with HOCl [30] they cannot be used for these investigations. Thus, inert buffers such as phosphate (with +V as the maximum oxidation state of phosphorus in phosphate) and/or acetate have to be used. On the other hand, many MS ionization techniques such as ESI MS are negatively affected by the salt content of the sample [31]. To avoid ion suppression effects, the salt concentrations should not exceed low millimolar concentrations. In comparison to proteins which possess different functional groups with both, acidic and alkaline properties, the desalting of oxidized carbohydrates is much more challenging since labile groups, such as chloramines, are present [32]. This is discussed in more detail in the supplementary material. Since established desalting techniques did not work in our hand, we dealt with this problem in the following way: the reactions took place in moderately concentrated buffer using relatively concentrated carbohydrate solutions. After the desired reaction time, the solutions were diluted with organic solvent (acetonitrile) to reduce the salt content in the samples to an extent, which can be tolerated by ESI MS. Nevertheless, monitoring these reactions is difficult and will be discussed in the next chapters.

### 3.1 Investigation of glucosamine

**Fig. 2:**
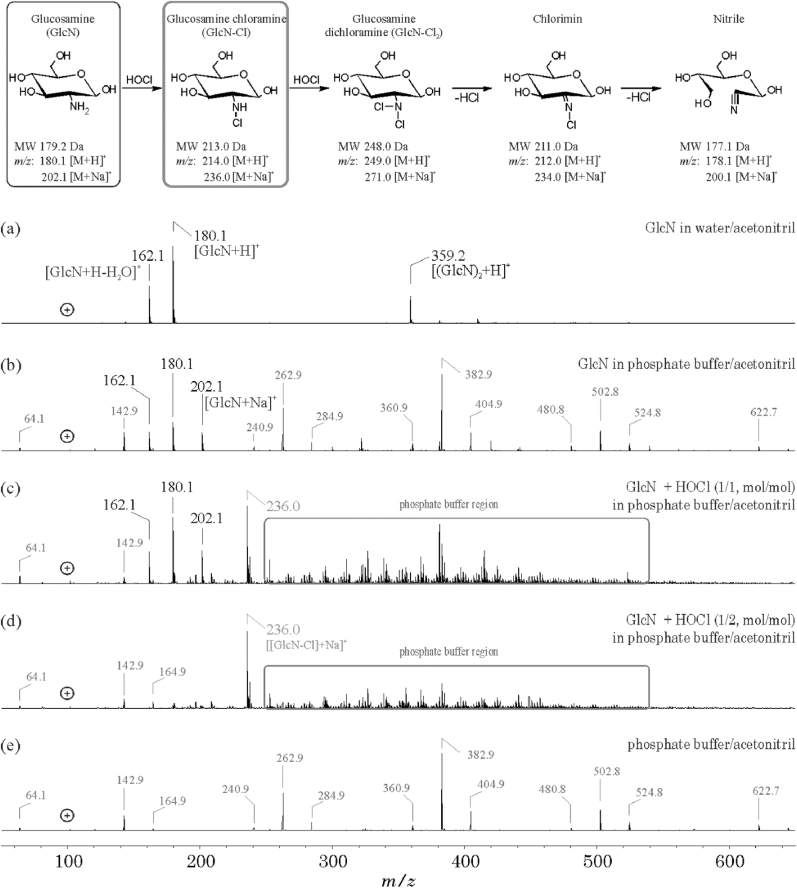
Positive ion electrospray ionization-ion trap (ESI-IT) mass spectra of glucosamine (GlcN, 250 μg/mL) either in water/acetonitrile (1/1, *v*/*v* (a)) or 20 mmol/L phosphate buffer/acetonitrile (1/1, v/v, (b)). The *m/z* values 162.1, 180.1 and 202.1 in the spectrum of pure GlcN (a and b) correspond to [GlcN + H–H_2_O]^+^, [GlcN + H]^+^ and [GlcN + Na]^+^, respectively. HOCl-induced oxidation/chlorination of GlcN was performed by using an equimolar ratio (c) or a twofold molar excess of HOCl (d). The arising of the GlcN-chloramine (GlcN-Cl, *m/z* 236.0, as the sodium adduct) is obvious from both spectra (c and d). The “background” stemming from the presence of the phosphate buffer is illustrated in (e). The majority of the observed peaks are caused by oligomers of phosphate, for instance, the trimer (H_6_P_3_O_12_Na_4_) at *m/z* 382.9. Potential reaction products between GlcN and HOCl are shown at the top of the figure.

Our first aim was the monitoring of the reaction between GlcN or GlcNAc and HOCl since this is a very simple reaction and should give information whether MS detection of N–Cl linkages in carbohydrates would be possible at all. This reaction could be successfully monitored by ESI MS in positive ion mode after direct infusion (Fig. 2).

Additionally, Fig. 2 illustrates the impact of the salt content on the detectability of the pure GlcN. Compared with the pure monosaccharide (250 μg/mL) in water/acetonitrile (1/1, *v*/*v*) (Fig. 2a), the spectrum was much more complex if 20 mmol/L phosphate buffer/acetonitrile (1/1, *v*/*v*) was used as the solvent (Fig. 2b). GlcN (MW 179.2) in water yielded two and in buffer three peaks at *m/z* 162.1, 180.1 and 202.1 corresponding to [GlcN + H–H_2_O]^+^, [GlcN + H]^+^ and [GlcN + Na]^+^, respectively. The *m/z* ratios labeled in grey are stemming from the buffer solution (background, Fig. 2e) and can be explained by phosphate oligomers, which are generated in the gas phase. It is important to note that the removal of salts is very difficult in the case of these carbohydrates of interest. The corresponding information is summarized in the Supplementary Information.

Since GlcN reacts rapidly with HOCl (using a ratio of 1:2), neither a long-term incubation nor a high molar excess of HOCl was necessary to yield a complete conversion into the corresponding GlcN monochloramine (GlcN-Cl). This is in line with the data given in a very recent article [19]. However, more effective chlorinating agents were used in this study because the yields were poor if the reagent HOCl was used. This is a convincing proof of the moderate yields that we obtained and a strong indication that an excess of HOCl must be used.

An equivalent molar ratio of GlcN and HOCl in phosphate buffer followed by a short-term mixing (1 min) led to an approximate 1:1 ratio between GlcN and GlcN-Cl. In addition to the peaks of the educt, an additional peak at *m/z* 236.0 [GlcN-Cl]+Na^+^ (Fig. 2c) could be detected which shows the typical isotopic pattern of chlorine (3:1 = ^35^Cl:^37^Cl). The use of an 1:2 M excess (GlcN:HOCl) led - under otherwise identical oxidation conditions - to a complete conversion of GlcN into GlcN-Cl (Fig. 2d). It might be surprising that there was no dichloramine (GlcN-Cl_2_) detectable even if a significant excess of HOCl was used (data not shown). However, it is known from previous work that dichloramines represent transient products and eliminate HCl under generation of the chlorimine and/or the nitrile as the main products [33]. Neither these two products nor products generated by opening of the hexose ring could be detected by ESI-IT MS - It is also remarkable that it was not possible to monitor chloramines if the chlorination is performed in the presence of trichloroisocyanuric acid (TCA, data not shown), which is an established and powerful chlorination agent [34]. However, we did not investigate this reaction in more detail since the GlcN served only as a model compound in order to ensure that the expected primary reaction products are detectable. Finally, the chlorination of carbohydrates of the GlcN type is much slower in comparison to typical amino groups (for instance, in α-amino acids such as glycine) where second order rate constants (k) of about 10^5^ M^−1^ s^−1^ were found [35]. This result also agrees with previous investigations [36] which reported a second order rate constant of 3.1 × 10^5^ M^−1^ s^−1^ for the conversion of GlcN into GlcN-Cl. The same authors [36] also indicated that the subsequent reaction of the GlcN-Cl into the GlcN-Cl_2_ was orders of magnitude slower (about 9 M^−1^ s^−1^ (at 37 °C)). This might be one reason why we detected exclusively the monochloramine (GlcN-Cl).

### 3.2 Investigation of N-acetylglucosamine (GlcNAc)

Since GlcNAc is one of the repeating units of the HA polysaccharide and the *N*-acetyl group is considered as the site of HA where the degradation or modification of HA is initiated [15], this carbohydrate is a more convincing model monosaccharide compared to GlcN. It has been already demonstrated by GC MS [20] that arabinose and arabinonic acid are the main products of HOCl oxidation of GlcNAc, i.e. a ring contraction occurs under these conditions. As expected, the reactivity between GlcNAc and HOCl is orders of magnitude slower compared to GlcN (about 0.01 compared to 3.1 × 10^5^ M^−1^ s^−1^ at neutral pH) [37] which will be illustrated in more detail by kinetic data obtained by UV spectroscopy (see below).

**Fig. 3:**
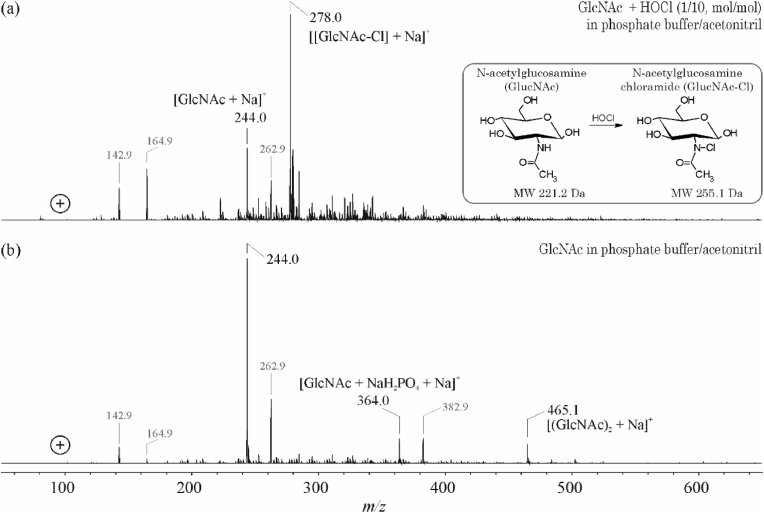
Positive ion ESI-IT mass spectra of *N*-acetylglucosamine (GlcNAc, (a)) and after the oxidation/chlorination with a tenfold molar excess of HOCl after 5 h incubation (b). The only observed product was the chloramide of GlcNAc (GlcNAc-Cl, *m/z* 287.0) besides the educt at *m/z* 244.0. The third peak at *m/z* 465.1 corresponds to a gas phase generated sodiated dimer of the GlcNAc educt.

It is obvious from the positive ion ESI-MS measurements (Fig. 3) that the buffer (grey labeled *m/z* values) has a considerable impact on the achievable signal-to-noise (S/N) ratio of the GlcNAc spectra (*m/z* 244.1, according to the Na^+^ adduct of GlcNAc).

Nevertheless, the expected *m/z* ratio of the chlorinated product was clearly detectable (Fig. 3a). After incubation of the carbohydrate with a tenfold molar excess of HOCl, an additional peak at *m/z* 278.1 [GlcNAc-Cl + Na]^+^ could be observed, which showed the typical chlorine isotopic pattern and, thus, corresponds to the expected chlorinated product (GlcNAc-Cl). The peak at *m/z* 465.2 was identified as a gas phase generated dimer of GlcNAc [2 × GlcNAc + Na]^+^ in the control sample, i.e. in the absence of HOCl under otherwise identical conditions (Fig. 3b).

No other reaction products were detectable, although it is commonly accepted that the stability of the GlcNAc-Cl is limited and that this transient product decays under generation of an *N*-centered radical, which subsequently rearranges into a *C*-centered radical [15]. It is also remarkable that there is still some unmodified N-acetylglucosamine left although the reaction was performed with a tenfold molar excess of HOCl. Nevertheless, this agrees with [19], where it is explicitly stated, that HOCl does not represent an efficient chlorinating agent. The yield of chlorinated products is much higher if TCA instead of HOCl is used (data not shown) but this agent has no physiological relevance at all.

**Fig. 4:**
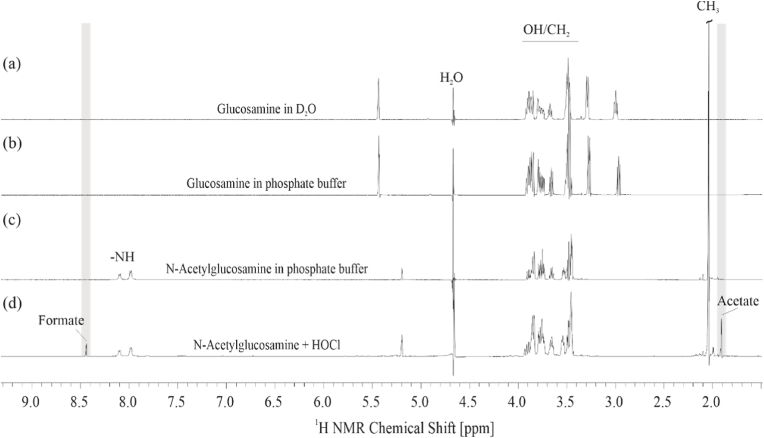
700 MHz ^1^H NMR spectra of GlcN (a,b) and GlcNAc (c,d) in the absence and the presence of an excess of HOCl. While there are no significant changes in the case of GlcN, the generation of other products (such as acetate and formate) is obvious when GlcNAc is incubated with HOCl. Acetate (grey bar, 1.90 ppm) results from the cleavage of the N-acetyl side chain, while formate (grey bar, 8.44 ppm) indicates the complete destruction of the carbohydrate ring. Note that the anomeric protons are barely detectable because they are close to the (attenuated) water resonance.

In contrast to the ESI mass spectrum, the ^1^H NMR spectrum (Fig. 4) gave a clear indication that there were (in addition to the chloroamide) also other products after the HOCl-induced oxidation of GlcNAc (Fig. 4d). First, a resonance at 1.90 ppm was detected which could be assigned to acetate [14] that is an already known product of the HOCl-induced degradation of *N*-acetylated carbohydrates [38]. Second, there was a weak resonance at 8.44 ppm, which could be assigned to formate as the final product (in addition to CO_2_ and H_2_O that are both unseen by ^1^H NMR) that arises from the complete degradation of the carbohydrate ring [16]. Although smaller carbohydrates could not be detected as such (presumably due to their low concentrations), formate generation indicates ring contraction, which was already discussed above [20].

It is very difficult to understand why smaller carbohydrate ring systems are not detectable by ESI-IT MS and why the GlcN oxidation (Fig. 4b) was not accompanied by formate generation since the formate is a comparably late degradation product of carbohydrates while CO_2_ is the very last product of sugar oxidation.

**Fig. 5:**
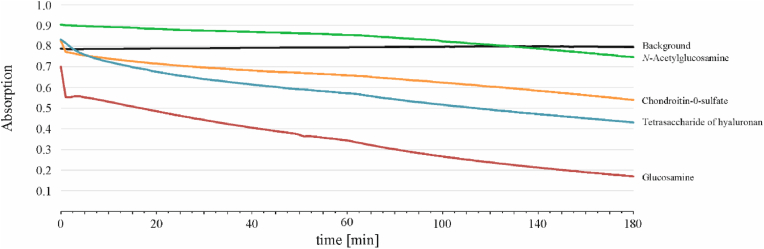
Monitoring of the HOCl consumption by different selected carbohydrates using UV spectroscopy. In order to compare the consumption of HOCl, its concentration (20 mmol/L) and volume (150 μL) were kept constant. The carbohydrate solutions (stock solution 1 mg/mL each) were calculated to obtain a molar ratio of 1:10 (sugar: HOCl). The total reaction volume of 500 μL, which was necessary for the used cuvettes, was made up with phosphate buffer (20 mmol/L). Kinetic measurements were performed for 180 min at room temperature.

Finally, it was very remarkable that there is still so much GlcNAc left although the reactant was treated with a tenfold molar excess of HOCl. In order to investigate this aspect in more detail, the consumption of HOCl was monitored by UV spectroscopy (Fig. 5). This is an established method to compare the reactivity of HOCl with different carbohydrates [39].

It is obvious from the reduced HOCl concentration that the relative reactivity decreased in the order GlcN > HA4 > C0S and, finally, GlcNAc as the carbohydrate with the lowest reactivity. It was not surprising that the GlcN possesses the highest reactivity because its amino function is very reactive and characterized by a similar second order rate constant as observed for taurine (about 3.5 × 10^3^ M^−1^ s^−1^ at pH 4.7) [40].

Remarkably, HA4 as well as C0S possess a higher reactivity than the GlcNAc. This is a clear indication that the glycosidic linkages are the preferred targets of HOCl, since glucuronic acid (GlcA), one of the repeating units of the relevant oligosaccharides, does not react with HOCl at all (data not shown). The glycosidic linkages are even more reactive than the N-acetyl side chain of GlcNAc. This aspect will now be verified by showing that the treatment of selected glycosaminoglycan oligosaccharides with HOCl leads to the release of the related monosaccharides. We will focus on the tetrasaccharide of HA because this compound is (comparably) readily available and in reasonable yields by enzymatic digestion of the HA polysaccharide [24]. Another problem with the investigation of oligosaccharides of the CS type would be the presence of the double bond (introduced by the enzymatic digestion) and, last but not least, the presence of the sulfate residue. Since the sulfate residues may be lost under MS conditions [41], this would further complicate the conditions.

### 3.3 Investigation of selected oligosaccharides of GAG

In addition to the reactive, functional groups which are present in the so far investigated monosaccharides, the tetrasaccharide of hyaluronan (HA4) contains three glycosidic linkages (see Fig. 1) which are also reactive towards HOCl - as evidenced by the UV spectroscopic experiments (vide supra). In contrast, the chondroitin sulfate (CS)-derived disaccharide, which can be generated by chondroitinase ABC digestion of the polysaccharide [42], contains only a single glycosidic linkage.

**Fig. 6:**
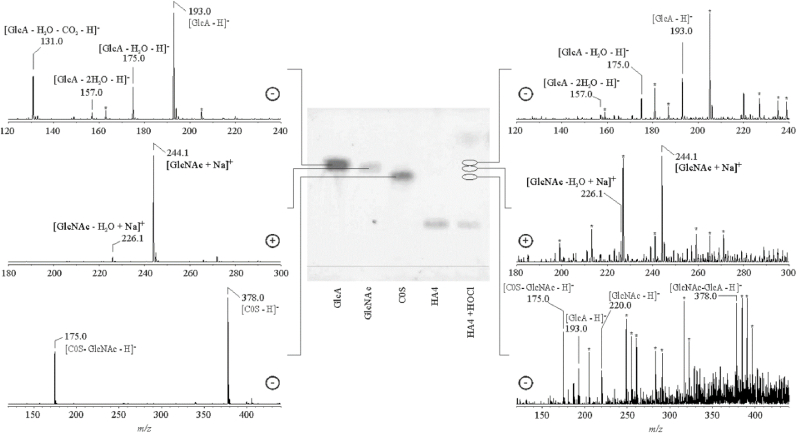
In the center of the figure, a developed TLC plate with different selected carbohydrates is shown. Only the sample at the right (HA4) was treated with an excess of HOCl whereas all other lanes just show isolated carbohydrates as the controls. From the left: glucuronic acid (GlcA), N-acetylglucosamine (GlcNAc), the disaccharide of chondroitin (C0S) and the HA tetrasaccharide (HA4). The positions, at which the corresponding ESI IT mass spectra of the reaction products were acquired, are indicated. Peaks of standards and reaction products are explicitly marked in the spectra. Note that cleavage of glycosidic linkages is obviously the most relevant reaction leading to the generation of monosaccharides. Peaks stemming from impurities from the TLC plates and/or the TLC conditions are marked by asterisks.

In Fig. 6 the negative and positive ion ESI-IT mass spectra of the HA tetrasaccharide subsequent to the reaction with a tenfold molar excess of HOCl (5 h, 37 °C) are shown at the right. These spectra were obtained subsequent to the TLC separation of the reaction mixture. The spectra of GlcA, GlcNAc and C0S as selected reference compounds, which were assigned according to their chromatographic (and characteristic MS) properties, are shown at the left.

Spectra at the left represent the different carbohydrate standards. Although the individual carbohydrates show only slightly different Rf values, their differentiation by MS is much easier. All compounds are detected with different sensitivities in the positive or negative ion mode. In the case of C0S in-source fragmentation can be observed, which leads to the neutral loss of the GlcNAc unit yielding the C0S-GlcNAc fragment, which corresponds to the GlcA unit of C0S. The GlcNAc unit would be much more sensitively detectable in the positive ion mode as the corresponding H^+^ or Na^+^ adduct. This also indicates the necessity of prior separation e.g. by TLC before ESI-MS analysis in order to ensure no false positive identification of in-source generated fragment ions as potential cleavage products.

The mechanism by which cleavage of the glycosidic linkage occurs at these conditions is unknown: on the one hand, it is well-known that the ESI spray conditions lead to a considerable decrease in the pH value which is presumably sufficient to hydrolyze (at least partially) the glycosidic linkages [43]. This process is, however, of limited relevance because otherwise there should be also fragments in the control sample. On the other hand, it is assumed that HOCl leads to the depolymerization of the HA polysaccharide [12] - although this reaction is still debated [44,45].

It is obvious from the mass spectra at the right that the lane of the HA4 (subsequent to reaction with HOCl) contains different mono- as well as disaccharides. Both, GlcA (*m/z* 193.0) and GlcNAc (*m/z* 244.1) are clearly detectable by MS due to its higher sensitivity - although the spot intensities on the TLC plate are poor. In any case, water loss is more pronounced in the HOCl-treated sample compared to the standards on the left; this is not surprising because the acidic conditions at TLC conditions favor the water elimination due to protonation [43]. The amount of the released disaccharide (*m/z* 378.0) is very poor - as reflected by the extent of impurities which were already previously described to be released from the TLC plate [46].

It is surprising that there are no indications of the generation of chloramides. Chloramides were recently suggested as the main products if high molecular weight hyaluronan reacts with HOCl - and particularly with trichloroisocyanuric acid (TCA) as the chlorinating agent. It was also indicated [19] that HOCl primarily leads to depolymerization of the HA polysaccharide whereas TCA generates the chloramide but avoids chain scission. We failed to reproduce this experiment: after treatment of HA4 with TCA there was no chloramide generation but only fragmentation either under loss of the GlcNAc or GlcA occurred (data not shown).

This finding is in contrast to previous reports where the N-acetyl group was indicated to possess the most pronounced reactivity towards HOCl [15]. Nevertheless, this is in line with the finding that HOCl leads to the depolymerization of the HA polysaccharide [12]. We have just initiated additional studies to exclude potential effects of trace amounts of catalytically active transition metals such as iron, i.e. Fenton chemistry [47]. We will also investigate whether Cl_2_O [48] is likely to contribute to the observed effects. Cl_2_O is increasingly assumed to play (in addition to Cl_2_ and HOCl) a major role as oxidant but was so far (to our best knowledge) not at all investigated in the field of GAG degradation.

## 4 Conclusions

We have shown that N-chlorinated carbohydrates can be detected by soft-ionization ESI MS. Both, chloroamines (from glucosamine) as well as chloramides (from N-acetylglucosamine) were readily detectable -at least in the case of the monosaccharides. However, we failed to detect such chlorinated products if HOCl reacts with the tetrasaccharide of hyaluronan or the disaccharide of chondroitin. Here, non-modified monosaccharides were the exclusive products. Although further efforts are necessary to clarify the related mechanisms, this is a clear indication that the glycosidic linkages represent the most important targets of HOCl and react more readily than the N-acetyl side chains. This has been also confirmed by measuring the HOCl consumption by different carbohydrates at which both, the tetrasaccharide of hyaluronan and the disaccharide of chondroitin, consumed the HOCl faster than N-acetylglucosamine. Thus, the glycosidic linkages are more reactive than the amino- or amide side groups. This emphasizes the role of low molecular weight fragments of HA and other glycosaminoglycans as markers of oxidative stress.

## Declaration of competing interest

The authors declare that they have no known competing financial interests or personal relationships that could have appeared to influence the work reported in this paper.

## References

[1] Wright H.L., Moots R.J., Bucknall R.C., Edwards S.W. (2010). Neutrophil function in inflammation and inflammatory diseases. Rheumatology.

[2] Boissier M.C., Semerano L., Challal S., Saidenberg-Kermanac'h N., Falgarone G. (2012). Rheumatoid arthritis: from autoimmunity to synovitis and joint destruction. J. Autoimmun..

[3] Schiller J., Fuchs B., Arnhold J., Arnold K. (2003). Contribution of reactive oxygen species to cartilage degradation in rheumatic diseases: molecular pathways, diagnosis and potential therapeutic strategies. Curr. Med. Chem..

[4] Weissmann G. (2004). Pathogenesis of rheumatoid arthritis. J. Clin. Rheumatol..

[5] Bala A., Mondal C., Haldar P.K., Khandelwal B. (2017). Oxidative stress in inflammatory cells of patient with rheumatoid arthritis: clinical efficacy of dietary antioxidants. Inflammopharmacology.

[6] Schiller J., Benard S., Reichl S., Arnhold J., Arnold K. (2000). Cartilage degradation by stimulated human neutrophils: reactive oxygen species decrease markedly the activity of proteolytic enzymes. Chem. Biol..

[7] Wright H.L., Moots R.J., Edwards S.W. (2014). The multifactorial role of neutrophils in rheumatoid arthritis. Nat. Rev. Rheumatol..

[8] Arnhold J., Flemmig J. (2010). Human myeloperoxidase in innate and acquired immunity. Arch. Biochem. Biophys..

[9] Strzepa A., Pritchard K.A., Dittel B.N. (2017). Myeloperoxidase: a new player in autoimmunity. Cell. Immunol..

[10] Wang G., Nauseef W.M. (2015). Salt, chloride, bleach, and innate host defense. J. Leukoc. Biol..

[11] Katrantzis M., Baker M.S., Handley C.J., Lowther D.A. (1991). The oxidant hypochlorite (OCl-), a product of the myeloperoxidase system, degrades articular cartilage proteoglycan aggregate. Free Radic. Biol. Med..

[12] Baker M.S., Green S.P., Lowther D.A. (1989). Changes in the viscosity of hyaluronic acid after exposure to a myeloperoxidase-derived oxidant. Arthritis Rheum..

[13] Soltés L., Mendichi R., Kogan G., Schiller J., Stankovska M., Arnhold J. (2006). Degradative action of reactive oxygen species on hyaluronan. Biomacromolecules.

[14] Schiller J., Arnhold J., Gründer W., Arnold K. (1994). The action of hypochlorous acid on polymeric components of cartilage. Biol. Chem. Hoppe Seyler.

[15] Hawkins C.L., Davies M.J. (1998). Degradation of hyaluronic acid, poly- and monosaccharides, and model compounds by hypochlorite: evidence for radical intermediates and fragmentation. Free Radic. Biol. Med..

[16] Schiller J., Arnhold J., Schwinn J., Sprinz H., Brede O., Arnold K. (1998). Reactivity of cartilage and selected carbohydrates with hydroxyl radicals: an NMR study to detect degradation products. Free Radic. Res..

[17] Akeel A., Sibanda S., Martin S.W., Paterson A.W., Parsons B.J. (2013). Chlorination and oxidation of heparin and hyaluronan by hypochlorous acid and hypochlorite anions: effect of sulfate groups on reaction pathways and kinetics. Free Radic. Biol. Med..

[18] Corsaro M.M., Pietraforte D., Di Lorenzo A.S., Minetti M., Marino G. (2004). Reaction of peroxynitrite with hyaluronan and related saccharides. Free Radic. Res..

[19] Buffa R., Hermannová M., Sojka M., Svozil V., Šulc P., Halamková P., Pospíšilová M., Krejčí H., Velebný V. (2020). Hyaluronic acid chloramide - synthesis, chemical structure, stability and analysis of antimicrobials. Carbohydr. Polym..

[20] Jahn M., Baynes J.W., Spiteller G. (1999). The reaction of hyaluronic acid and its monomers, glucuronic acid and N-acetylglucosamine, with reactive oxygen species. Carbohydr. Res..

[21] Kettle A.J., Albrett A.M., Chapman A.L., Dickerhof N., Forbes L.V., Khalilova I., Turner R. (2014). Measuring chlorine bleach in biology and medicine. Biochim. Biophys. Acta.

[22] Cai M.Q., Feng L., Jiang J., Qi F., Zhang L.Q. (2013). Reaction kinetics and transformation of antipyrine chlorination with free chlorine. Water Res..

[23] Cheng H., Song D., Chang Y., Liu H., Qu J. (2015). Chlorination of tramadol: reaction kinetics, mechanism and genotoxicity evaluation. Chemosphere.

[24] Köhling S., Künze G., Lemmnitzer K., Bermudez M., Wolber G., Schiller J., Huster D., Rademann J. (2016). Chemoenzymatic synthesis of nonasulfated tetrahyaluronan with a paramagnetic tag for studying its complex with interleukin-10. Chemistry.

[25] Morris J.C. (1966). The acid ionization constant of HOCl from 5° to 35. J. Phys. Chem..

[26] Settembre C., Fraldi A., Medina D.L., Ballabio A. (2013). Signals from the lysosome: a control centre for cellular clearance and energy metabolism. Nat. Rev. Mol. Cell Biol..

[27] Nimptsch K., Süss R., Riemer T., Nimptsch A., Schnabelrauch M., Schiller J. (2010). Differently complex oligosaccharides can be easily identified by matrix-assisted laser desorption and ionization time-of-flight mass spectrometry directly from a standard thin-layer chromatography plate. J. Chromatogr. A.

[28] Grisham M.B., Jefferson M.M., Melton D.F., Thomas E.L. (1984). Chlorination of endogenous amines by isolated neutrophils. Ammonia-dependent bactericidal, cytotoxic, and cytolytic activities of the chloramines. J. Biol. Chem..

[29] Zheng G., Price W.S. (2010). Solvent signal suppression in NMR. Prog. Nucl. Magn. Reson. Spectrosc..

[30] Prütz W.A. (1996). Hypochlorous acid interactions with thiols, nucleotides, DNA, and other biological substrates. Arch. Biochem. Biophys..

[31] Canarelli S., Fisch I., Freitag R. (2002). On-line microdialysis of proteins with high-salt buffers for direct coupling of electrospray ionization mass spectrometry and liquid chromatography. J. Chromatogr. A.

[32] Raftery M.J. (2007). Detection and characterization of N-alpha-chloramines by electrospray tandem mass spectrometry. Anal. Biochem..

[33] Richter G., Schober C., Süss R., Fuchs B., Birkemeyer C., Schiller J. (2008). Comparison of the positive and negative ion electrospray ionization and matrix-assisted laser desorption ionization-time-of-flight mass spectra of the reaction products of phosphatidylethanolamines and hypochlorous acid. Anal. Biochem..

[34] Hiegel G.A., Hogenauer T.J., Lewis J.C. (2005). Preparation of N-chloroamides using trichloroisocyanuric acid. Synth. Commun..

[35] Pattison D.I., Davies M.J. (2001). Absolute rate constants for the reaction of hypochlorous acid with protein side chains and peptide bonds. Chem. Res. Toxicol..

[36] Rees M.D., Pattison D.I., Davies M.J. (2005). Oxidation of heparan sulphate by hypochlorite: role of N-chloro derivatives and dichloramine-dependent fragmentation. Biochem. J..

[37] Panasenko O.M., Gorudko I.V., Sokolov A.V. (2013). Hypochlorous acid as a precursor of free radicals in living systems. Biochemistry (Mosc.).

[38] Flugge L.A., Miller-Deist L.A., Petillo P.A. (1999). Towards a molecular understanding of arthritis. Chem. Biol..

[39] Schiller J., Arnhold J., Arnold K. (1995). Action of hypochlorous acid on polymeric components of cartilage. Use of 13C NMR spectroscopy. Z. Naturforsch. C Biosci..

[40] Marquez L.A., Dunford H.B. (1994). Chlorination of taurine by myeloperoxidase. Kinetic evidence for an enzyme-bound intermediate. J. Biol. Chem..

[41] Lemmnitzer K., Köhling S., Freyse J., Rademann J., Schiller J. (2021). Characterization of defined sulfated heparin-like oligosaccharides by electrospray ionization ion trap mass spectrometry. J. Mass Spectrom..

[42] Schiller J., Arnhold J., Benard S., Reichl S., Arnold K. (1999). Cartilage degradation by hyaluronate lyase and chondroitin ABC lyase: a MALDI-TOF mas spectrometric study. Carbohydr. Res..

[43] Prebyl B.S., Kaczmarek C., Tuinman A.A., Baker D.C. (2003). Characterizing the electrospray-ionization mass spectral fragmentation pattern of enzymatically derived hyaluronic acid oligomers. Carbohydr. Res..

[44] Saari H., Konttinen Y.T., Friman C., Sorsa T. (1993). Differential effects of reactive oxygen species on native synovial fluid and purified human umbilical cord hyaluronate. Inflammation.

[45] Duan J., Kasper D.L. (2011). Oxidative depolymerization of polysaccharides by reactive oxygen/nitrogen species. Glycobiology.

[46] Morlock G.E. (2014). Background mass signals in TLC/HPTLC-ESI-MS and practical advices for use of the TLC-MS interface. J. Liq. Chromatogr. Relat. Technol..

[47] Zhao X., Yang B., Li L., Zhang F., Linhardt R.J. (2013). On-line separation and characterization of hyaluronan oligosaccharides derived from radical depolymerization. Carbohydr. Polym..

[48] Sivey J.D., McCullough C.E., Roberts A.L. (2010). Chlorine monoxide (Cl(2)O) and molecular chlorine (Cl(2)) as active chlorinating agents in reaction of dimethenamid with aqueous free chlorine. Environ. Sci. Technol..

