# Supplementary material for: Mass spectrometric investigations of the action of hypochlorous acid on monomeric and oligomeric components of glycosaminoglycans
Source: Biochem Biophys Rep. 2023 Mar 2;34:101448. doi: 10.1016/j.bbrep.2023.101448 (PMC10006533; doi:10.1016/j.bbrep.2023.101448)
Supplement: Multimedia component 1 [file mmc1.docx]

**Supplementary Material**

Jenny Leopold, Patricia Prabutzki, Ariane Nimptsch and Jürgen Schiller

Mass spectrometric Investigations of the Action of Hypochlorous Acid on monomeric and oligomeric Components of Glycosaminoglycans

LC/MS is much more established and more widely used than TLC/MS. Nevertheless, we used for the investigation of oxidatively modified GAG mono- and oligosaccharides exclusively TLC/MS. We would like to report here shortly which results can be obtained by LC/MS with the same samples.

**Methods**

**High performance liquid chromatography / mass spectrometry (HPLC/MS)**

High-performance liquid chromatography (HPLC) measurements were performed with a Ultimate 3,000 system (Thermo Fisher Scientific GmbH, Dreieich, Germany) coupled to an amaZon SL (Bruker Daltonics, Bremen, Germany) ion trap (IT) mass spectrometer equipped with an electrospray ionization (ESI) source. Two different hydrophilic interaction liquid chromatography (HILIC) columns were tested [1]. First, the iHILIC Fusion(+) (50×2.1 mm, 3.5 µm, HILICON AB, Umea, Sweden) and second, a Shodex SUGAR KS-801 (300×8mm, 6µm, Resonac Europe GmbH, Munich, Germany) [2].

The Shodex SUGAR KS-801 was used either with pure water or with a maximum of 20% ethanol as eluent. 10 µL of GAG standards (c = 10 µg/ml in acetonitrile) were separated by an isocratic elution either with 100% water or ethanol/water (2/8; v/v) and different flow rates and conditions (see text). With the iHILIC Fusion(+), 10 µL of GAG standards (c = 10 µg/ml in acetonitrile) either as isolated compounds or subsequent to reaction with HOCl were separated using the following conditions: eluent A was 50 mM ammonium formate in water containing 0.1% formic acid, and eluent B was pure acetonitrile containing 0.1% formic acid. The separation was achieved by a flow rate of 0.15 ml/min and the following gradient: 0 min: 20% B; 2 min: 56% B; 3 min: 60% B; 5 min: 60% B, 5.1 min: 20% B; 6.5 min: 20% B. The column oven temperature was set to 50°C. ESI-IT MS conditions were the same as for the direct infusion as described before.

**Results and Discussion**

**Separation of GAG-derived carbohydrates and oxidation products**

In order to verify the TLC/MS results and with the aim to improve sensitivity, an HPLC/MS approach was established. One serious problem towards the use of LC for carbohydrate analysis is that the majority of columns do not tolerate high water contents or even salts. Thus, we compared an iHILIC and a Shodex SUGAR KS-801 column.

We started with the Shodex SUGAR column and used the recommended conditions of 100 % water as eluent, 50°C oven temperature and a flow rate of 0.4 ml/min. Since the water did not evaporate fast enough and water droplets were generated in the ion source, improved conditions had to be established. A different eluent consisting of 20% ethanol in water or acetonitrile was used. Since the Shodex column has to be operated with only small amounts of organic solvents, we introduced acetonitrile via a T-piece adapter right after the column, which allowed the separation of the carbohydrates in the column but overcame the problem with the poor evaporation in the ion source and enabled the "dilution" of potential background signals from the buffer system. Both eluent flow rates were varied between 0.2-0.4 ml/min. The column oven temperature was increased up to 70°C, which additionally improved the quality of the chromatographic separation. The flow rate was adjusted to 0.2 ml/ml to avoid poor evaporation of water-based eluents. The following parameters were established as the the optimum ones: 0.5 mg/ml sugar mix containing glucuronic acid, glucosamine, N-acetylglucosamine, chondroitin-0-sulfate disaccharide and hyaluronic acid tetrasaccharide; injection volume of 10 µl; Eluent A: 10% ethanol in water, eluent B using T piece adapter: pure sacetonitrile; flow rate of both eluents 0.4 ml/min; run time 25 min; column oven temperature: 70°C. The ESI-IT parameters were the same as in the case of the direct infusion measurements. Unfortunately, and from so far unknown reasons GlcN could not be detected at all, while the HA4 and C0S standards were not sufficiently resolved.

The latter problem could be overcome by using the iHILIC(+) column which enabled the baseline-separation of GlcNAc, C0S and HA4 standards in not more than 5 min (**Fig. S1**) which is verified by the different extracted ion chromatograms of *m/z* values 244.0 [GlcNAc+Na]^+^, 378.0 [C0S-H]^+^ and 387.1 [HA4-2H]^2+^, respectively. From so far unknown reasons, GlcN could neither be found in the GAG mix nor as isolated component. Since GlcNAc (and not GlcN) is one of the repeating units in HA (the compound of particular interest), we did not address the problem with the lacking GlcN detection in more detail.


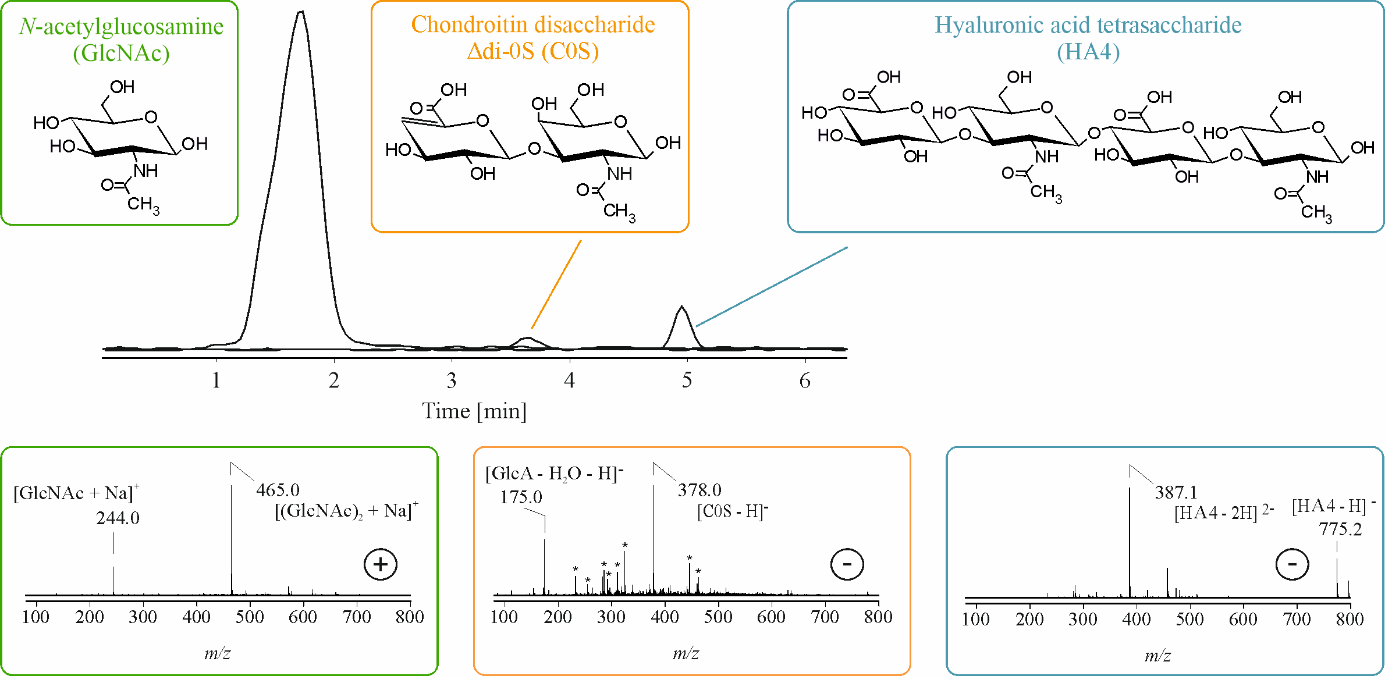


**Fig S1**: Extracted ion chromatograms and the corresponding spectra of GlcNAc (left), C0S (middle) and HA4 (right). The polarities of the measurements are indicated by either (+) or (-). Glucosamin (GlcN) could not be recovered after the LC-MS run. Please note that the negative ion spectrum of C0S, i.e. a disaccharide of 4,5-Δ-glucuronic acid and N-acetylgalactosamine, shows also significant amounts of glucuronic acid (GlcA).

In a second step, we investigated the detectability of chlorinated products of selected GAG monosaccharides by HPLC-MS. Thus, we oxidized GlcNAc (**Fig. S2**) and HA4 (**Fig. S3**) with HOCl as described before (cf. the Materials and Methods section of the original paper) and separated the obtained products with the developed HPLC method.


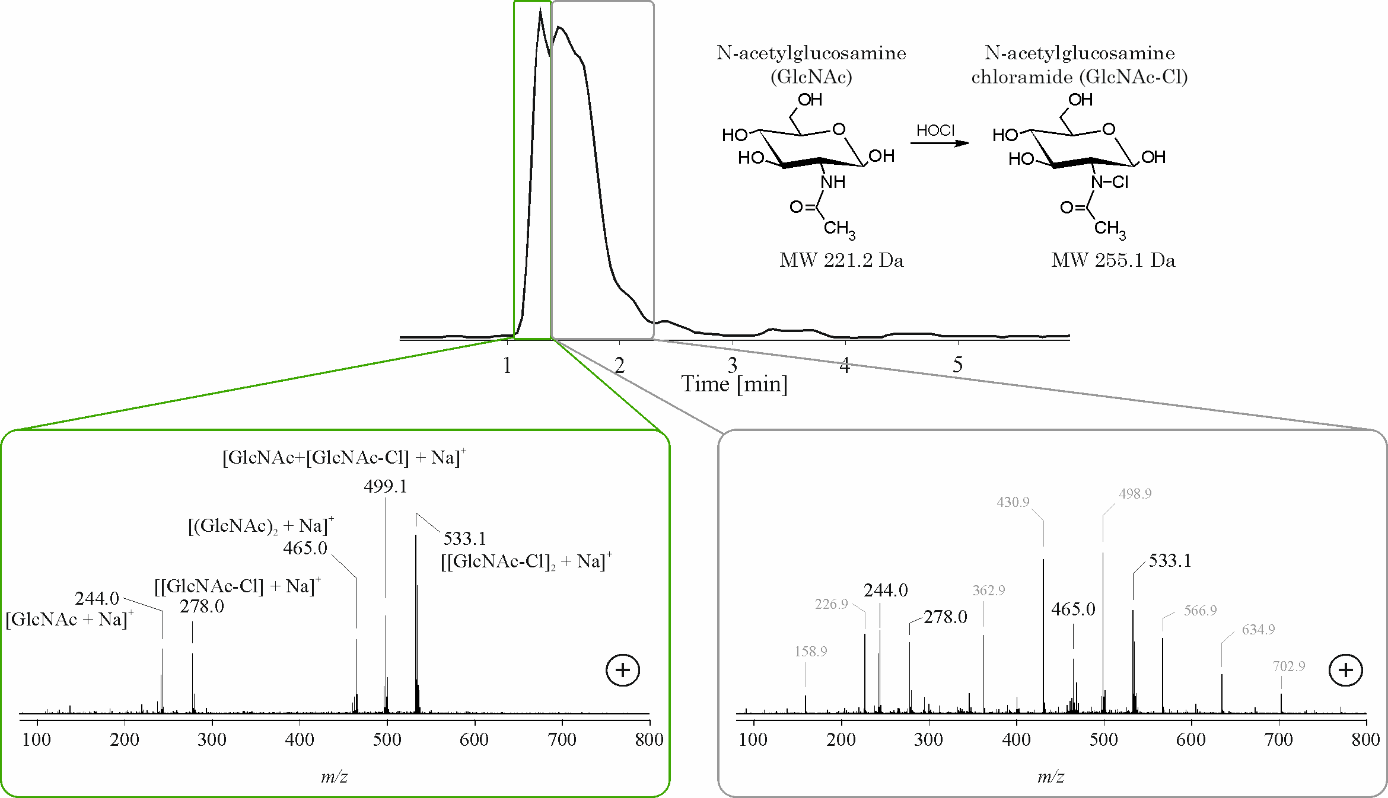


**Fig. S2**: Oxidation of isolated GlcNAc with HOCl and subsequent product analysis by HPLC-MS. Using the developed method the separation from native GlcNAc and the corresponding chloramine (GlcNAc-Cl) was not successful but the oxidation product was stable enough to pass the LC separation without decomposition. Retention times from both, GAG and buffer signals (marked by grey numbers; assigned in the original paper) overlaid each other from 1.4 - 2 min.

The LC-MS chromatogram of oxidized/chlorinated HA4 was more complex because four different peaks could be observed: peaks 1 and 3 are presumably caused by buffer and other background signals, which were barely resolved. Peak 4 could be identified as the non-oxidized HA4 standard (vide supra) while peak 2 could be assigned as the trisaccharide which was generated from HA4 by the cleavage of one glycosidic linkage. This compound was also detectable by TLC and will be discussed in more detail in the original paper.


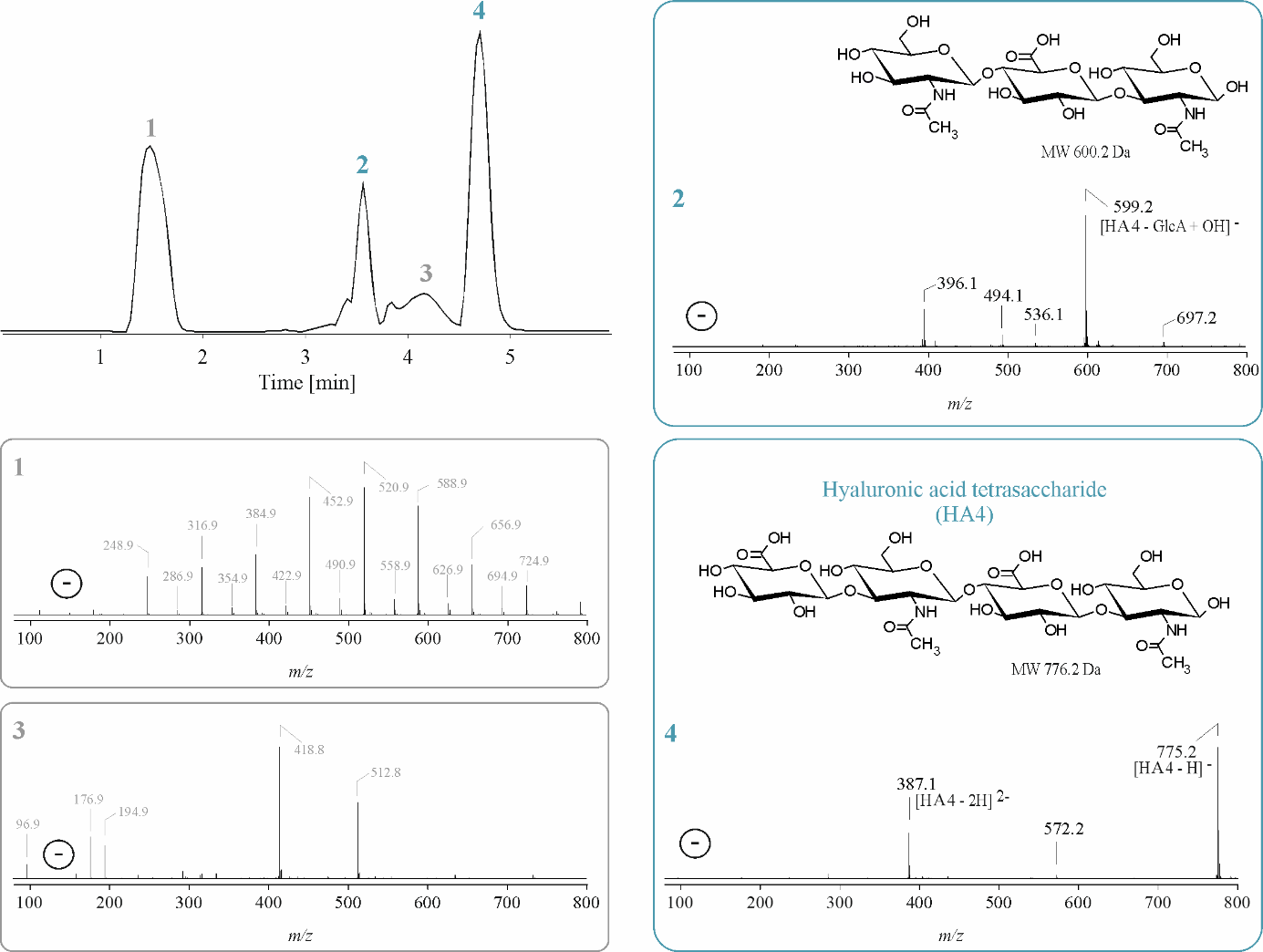


**Fig. S3**: Oxidation of isolated HA4 with HOCl and the subsequent product analysis by HPLC-MS. The developed method allowed the separation of the obtained products into four fractions. Peak 1 and 3 were identified as background signals stemming from the buffer solution. Peak 4 corresponds to the native HA4 educt. The most intense *m/z* signal (base peak) of peak 2 was identified as the trisaccharide by the cleavage of glucoronate from HA4. Oxidized/chlorinated products could not be detected. This leads to the assumption that the cleavage of the glycosidic linkages in glycosaminoglycans is more preferred than the formation of chloramines.

**Desalting of samples**

Although we used different types of chromatographic columns, we experienced that the desalting of the samples is challenging. There are two different widely used methods. First, desalting is normally performed by gel filtration, i.e. by the size difference between the analyte and the (buffer) salts such as phosphate. We were not successful with this attempt because the size difference between the salt and the mono- and oligosaccharides is not sufficient.

Second, many desalting processes (for instance the well-known "ZipTips") are based on alterations of the charge state of the analyte. This is, the salt (as a strong electrolyte) is always dissociated into ions but the charge of the analyte can be switched from positively charged to neutral or even negatively charged. This is possible with amino acids as well as proteins but we failed with the carbohydrates of interest. Since the oxidized (chlorinated) groups have limited stability towards changes of the pH value, desalting is accompanied by a loss of the functional groups of interest.

**References**

[1] Gill VL, Aich U, Rao S, Pohl C, Zaia J. Disaccharide analysis of glycosaminoglycans using hydrophilic interaction chromatography and mass spectrometry. Anal Chem. 2013;85:1138-1145. doi: 10.1021/ac3030448.

[2] Ke CL, Zeng XX. Ultrasonically assisted extraction and HPLC determination of chondroitin sulfate from fish heads. J Chem Soc Pak. 2012;34:557-564.
